# Supplementary material for: Vaccination with a Replication-Dead Murine Gammaherpesvirus Lacking Viral Pathogenesis Genes Inhibits WT Virus Infection
Source: bioRxiv. 2024 Nov 21:2024.11.20.624603. Preprint. [Version 1] doi: 10.1101/2024.11.20.624603 (PMC11722263; doi:10.1101/2024.11.20.624603)
Supplement: Supplement 1 [file media-1.pdf]

Supplemental Materials

Supplemental Methods

Restriction fragment length polymorphism: To confirm the integrity of the MHV68 BACmid after genetic manipulation, RFLP analysis was performed by *Hind*III restriction enzyme digestion of 1ug of BACmid DNA. Clone# 121 (RDV-50.stop), clone# 466 (RDV-50.stopΔM1-M4), and WT MHV68 BAC by overnight 37° C. Fragments were separated by 0.9% agarose electrophoresis at 30V for 20 hours.

Vβ4+ CD8 T cells analysis by flow cytometry: Spleens were processed using a tissue grinder and were treated to remove RBC and filtered as described previously prior to resuspension in 200 μl of PBS with 2% fetal bovine serum and blocked with TruStain fcX (BioLegend, San Diego, CA). T cell subsets were identified with antibodies against CD45 (dilution 1:200; clone 30-F11; BV510; cat# 563891), Vβ4+ (dilution 1:100; clone KT4; FITC; cat# 553365) purchased from BD Biosciences, Franklin Lakes, NJ and CD4 (dilution 1:400; clone GK1.5; PerCP-Cy5.5; cat# 100434), CD8 (dilution 1:400; clone 53-6.7; Pacific Blue; cat# 100725), CD62L (dilution 1:2000; clone MEL-14; PE; cat# 104408), CD44 (dilution 1:1000; clone IM7; APC; cat# 103012) purchased from BioLegend, San Diego, CA. The data was collected on a CytoFLEX flow cytometer (Beckman Coulter, Brea, CA) and analyzed using FlowJoX v10.10.0 (Treestar Inc., Ashland, OR). CD8+ T cells were first gated as lymphocytes based on forward and side scatter parameters, singlets, then live per exclusion of Alexa Fluor™ 700 NHS Ester uptake, followed by gating for Vβ4+ CD8+CD45+.

Table S1. Primers, gBlocks and antibodies used in this study

Mutation	gBlock[a]
**Mutant MHV68 generation**	
ORF50 locus<br>50_STOP_FS_gBLK_1	AGAAACCAGAAGGTGAGGTTTAATGCCAAAGTCCATAACAGGCATCCATGTGGGTACATA**TAG**TCTCACCACCTGATC**T**AAATATGCCATTGA**T**AAGAGTTGTCTA**G**ACCACAGACAGGCTGTTTCTTAGGGATAACAGGGTAATCGATTTATTCAACAAAGCCACGTTGTGTCTCAAAATCTCTGATGTTACATTGCACAAGATAAAAATATATCATCATGAACAATAAAACTGTCTGCTTACATAAACAGTAATACAAGGGGTGTTATGAGCCATATTCAACGGGAAACGTCTTGCTCGAGGCCGCGATTAAATTCCAACATGGATGCTGATTTATATGGGTATAAATGGGCTCGCGATAATGTCGGGCAATCAGGTGCGACAATCTATCGATTGTATGGGAAGCCCGATGCGCCAGAGTTGTTTCTGAAACATGGCAAAGGTAGCGTTGCCAATGATGTTACAGATGAGATGGTCAGACTAAACTGGCTGACGGAATTTATGCCTCTTCCGACCATCAAGCATTTTATCCGTACTCCTGATGATGCATGGTTACTCACCACTG
ORF50 locus<br>50_STOP_FS_gBLK_2	CTCCTGATGATGCATGGTTACTCACCACTGCGATCCCCGGGAAAACAGCATTCCAGGTATTAGAAGAATATCCTGATTCAGGTGAAAATATTGTTGATGCGCTGGCAGTGTTCCTGCGCCGGTTGCATTCGATTCCTGTTTGTAATTGTCCTTTTAACAGCGATCGCGTATTTCGTCTCGCTCAGGCGCAATCACGAATGAATAACGGTTTGGTTGATGCGAGTGATTTTGATGACGAGCGTAATGGCTGGCCTGTTGAACAAGTCTGGAAAGAAATGCATAAGCTTTTGCCATTCTCACCGGATTCAGTCGTCACTCATGGTGATTTCTCACTTGATAACCTTATTTTTGACGAGGGGAAATTAATAGGTTGTATTGATGTTGGACGAGTCGGAATCGCAGACCGATACCAGGATCTTGCCATCCTATGGAACTGCCTCGGTGAGTTTTCTCCTTCATTACAGAAACGGCTTTTTCAAAAATATGGTATTGATAATCCTGATATGAATAAATTGCAGTTTCATTTGATGCTCGATGAGTTTTTCTAATCAGAATTGGTTAATTGGTTGTAACACTGGCGGCATCCATGTGGGTACATA**TAG**TCTCACCACCTGATC**T**AAATATGCCATTGA**T**AAGAGTTGTCTA**G**ACCACAGACAGGCTGTTTCTGACTGCCCCATGTTCGGGGGTACAGCTACCTCAACCTCTG
M1-M4 deletion<br>M1_4DEL gBLK_1	CCTTTATTGTAAGGGTACTCTCATCACCAATGTAAATTAATATGTAGCAAACTTTGGTGTGGGAGTCCTACCCCTTTTGCTCCACAGGCCGCCCACGACCATCTAGCCTCCCACCAAAACAAGAACAGTTGCAGCTTTTGCTGTTTTCCTTAATATTTATCTTTGTGGTGTTCAGTCAGGCCTTGCTCTCAGTTTCTATGCCCCCAGGCTGGTTTCAAACACTGGTCAGAAGGCTATCTTTCTTGGTGGTTTCACTTCTAAACATGGGCCATTAAAAGGGAGGGAATTGGCATCATTGAGCAGCGGCGACCTAGGGATAACAGGGTAATCGATTTATTCAACAAAGCCACGTTGTGTCTCAAAATCTCTGATGTTACATTGCACAAGATAAAAATATATCATCATGAACAATAAAACTGTCTGCTTACATAAACAGTAATACAAGGGGTGTTATGAGCCATATTCAACGGGAAACGTCTTGCTCGAGGCCGCGATTAAATTCCAACATGGATGCTGATTTATATGGGTATAAATGGGCTCGCGATAATGTCGGGCAATCAGGTGCGACAATCTATCGATTGTATGGGAAGCCCGATGCGCCAGAGTTGTTTCTGAA

	ACATGGCAAAGGTAGCGTTGCCAATGATGTTACAGATGAGATGGTCAGACTAAACTGGCTGACGGAA<br>TTTATGCCTCTTCCGACCATCAAGCATTTTATCCGTACTCCTGATGATGCATGGTTACTCACCACTG
M1-M4 deletion<br>M1_4DEL gBLK_2	CTCCTGATGATGCATGGTTACTCACCACTGCGATCCCCGGGAAAACAGCATTCCAGGTATTAGAAGAA<br>TATCCTGATTCAGGTGAAAATATTGTTGATGCGCTGGCAGTGTTCCTGCGCCGGTTGCATTCGATTCCTG<br>TTTGTAATTGTCCTTTTAACAGCGATCGCGTATTTCGTCTCGCTCAGGCGCAATCACGAATGAATAACG<br>GTTTGGTTGATGCGAGTGATTTTGATGACGAGCGTAATGGCTGGCCTGTTGAACAAGTCTGGAAAGAA<br>ATGCATAAGCTTTTGCCATTCTCACCGGATTCAGTCGTCACTCATGGTGATTTCTCACTTGATAACCTTA<br>TTTTTGACGAGGGGAAATTAATAGGTTGTATTGATGTTGGACGAGTCGGAATCGCAGACCGATACCAG<br>GATCTTGCCATCCTATGGAACTGCCTCGGTGAGTTTTCTCCTTCATTACAGAAACGGCTTTTTCAAAAAT<br>ATGGTATTGATAATCCTGATATGAATAAATTGCAGTTTCATTTGATGCTCGATGAGTTTTTCTAATCAGA<br>ATTGGTTAATTGGTTGTAACACTGGCTTCTAAACATGGGCCATTAAAAGGGAGGGAATTGGCATCATT<br>GAGCAGCGGCGACCTTACATTCATCTGGGAATATGGTATTGAGATTTATGACTTTCTAGAATAACTGTA<br>CCCTGTTAAGTTTCAATTCCTTGTGGCCCTACCCCGAATCTCTATTAAAGGGTTAATAAAAATTACTCTC<br>AACAAAATCATGGCCAACTTCCACTTTTTCTGCGCAGTATTGGTGGGGATTGTGGGTGTAAATGGTGAC<br>AACATGTGCCACCATCTTCCTCAAATGCCCACACTAACATCTCCTAAATTTACACCACCAGTCAAAAG<br>TGGCACCACCCTGCAGCTCCGCTGTAGGCCAGGGTTCACACCAGGCGCA
**Mutation**	**Amplification Primers**
RDV-50.stop<br>50_STOP_FS_FOR	5′AGAAACCAGAAGGTGAGGTTTAATG
RDV-50.stop<br>50_STOP_FS_REV	5′CAGAGGTTGAGGTAGCTGTACCC
RDV-50.stopΔM1-M4<br>M1-4_DEL_FOR	5′-CACCAATGTAAATTAATATGTAGC
RDV-50.stopΔM1-M4<br>M1-4_DEL_REV	5′-AACCCTGGCCTACAGCGGAGCTGC
**Mutation**	**Sanger Sequencing Primers**
ORF50 locus<br>50_FSS_SEQ_FOR	5′ACAAATTTTACACAGCACCTGAAGC
ORF50 locus<br>50_FSS_SEQ_REV	5′ATGCCTCAACTTCTCTGGATATG

M1-M4 deletion<br>M1-4_SEQ_FOR	5′-CACCAATGTAAATTAATATGTAGC
M1-M4 deletion<br>M1-4_SEQ_REV	5'-GGTGGTGGTGCTCATGTCTGACG

[a]Bold letters - **TAG** and two **T's** were added, then a **G** was substituted for a T to obtain a *Xba*I site for screening which sufficiently disrupts the reading frame. Underlined-frameshift region

Supplemental Figure Legends

Supplementary Figure 1 Generation of recombinant RDV-50.stopΔM1-M4 MHV68 upon deletion of unique M gene locus. (a) Schematic describing the left end of WT MHV68 and the parental RDV-50.stop. M1- M4 genes and TMERs 6-8 (nucleotides 2022 to 9739) were deleted from RDV-50.stop to generate MHV68 RDV-50.stopΔM1-M4. '*' and '^' denote HindIII digestion sites. '*' indicates the site lost with the deletion of 2022 to 9739 bp. Figure made with BioRender. **(b)** In the HindIII restriction digest, WT MHV68 and RDV-50.stop has the expected 6156 bp fragment, while the RDV-50.stopΔM1-M4 genome has the expected loss of the 6156 bp fragment and gain of a 3277 bp fragment, indicated by red arrows.

Supplementary Figure 2 RDV-50.stopΔM1-M4 generates virus-specific CD8 T cell responses but does not induce M1 driven Vβ4+ CD8 T cells upon a prime-boost regimen in C57BL/6 mice. C57BL/6 mice were either sham-vaccinated or prime-boost vaccinated IP with $1x10^6$ PFU RDV-50.stopΔM1-M4 or RDV-50.stop. Naïve mice were age-matched, non-vaccinated controls. **(a)** Total p79-tetramer+ CD8 T cells per spleen of individual mice at d28 post-boost. Percentage of p79-dextramer+ CD8 T cells with markers of **(b)** short-lived effector cell (SLEC, KLRG1+CD127-) and **(c)** memory precursor effector cell subsets (MPEC, KLRG1-CD127+). **(d)** MPECs were further delineated into CD62L- effector and CD62L+ central MPECs for p79- dextramer+ CD8 T cells. **(e)** Percentage of Vβ4+ CD8 T cells in pooled spleens of mice with RDV-50.stop or RDV-50.stopΔM-M4 vaccination or infection with WT MHV68. For **(a-d)**, symbols represent individual mice, (N=5), and for **(e)** symbols represent pooled spleens from 5 mice.; bars and whiskers are mean +/- SD. *, p<0.05; **, p<0.01; ***, p<0.001 in Sidak's multiple comparisons test of one-way ANOVA **(a)**; in two-tailed unpaired t test **(b-d)** between the indicated groups.

Supplementary Figure 3 Vaccination with RDV-50.stopΔM1-M4 reduces acute replication, latency and reactivation in C57BL/6 mice. C57BL/6 mice either sham-vaccinated or prime-boost vaccinated IP with $1x10^6$ PFU of RDV-50.stopΔM1-M4 or RDV-50.stop followed by IP challenge with $5x10^3$ PFU of WT MHV68 at d28 post-boost. **(a)** Acute replication at d7 post-challenge determined by measuring infectious particles per ml lung homogenate. (N=5 mice per experiment); bars and whiskers represent mean +/- SD. ****, p<0.0001 in Sidak's multiple comparisons test of one-way ANOVA between the indicated groups. **(b)** The frequency of latency determined by limiting dilution nested

PCR of intact splenocytes for the viral genome at day d28 post-boost. **(c)** The frequency of explant reactivation determined by limiting dilution coculture of intact viable splenocytes on a monolayer of primary MEFs d28 post-boost. Disrupted splenocytes plated in parallel did not detect preformed infectious virus in the vaccinated animals. For **(b-c),** symbols represent pool of five individual mice per experiment.

Supplementary Figure 4 Evaluation of T cell response to MHV68 in the spleens of vaccinated mice at seventeen days post-challenge with WT virus. C57BL/6 mice were either sham-vaccinated or prime-boost vaccinated IP with $1x10^6$ PFU RDV-50.stopΔM1-M4 or RDV-50.stop followed by IN challenge with $1x10^3$ PFU WT MHV68 at d28 post-boost and analyzed d17 post-challenge. **(a)** Total p79-dextramer+ CD8 T cells per spleen of individual mice with the indicated vaccination regimen. Percentage of p79-dextramer+ CD8 T cells with markers of **(b)** short-lived effector cell (SLEC, $KLRG1^+CD127^-$) and **(c)** memory precursor effector cell subsets (MPEC, $KLRG1^-CD127^+$). **(d)** MPECs were further delineated into $CD62L^-$ effector and $CD62L^+$ central MPECs for p79-dextramer+ CD8 T cells. For each graph, symbols represent individual mice, (N=3-5); bars and whiskers are mean +/- SD. **, $p<0.01$ in Sidak's multiple comparisons test of one-way ANOVA between the indicated groups.

Supplemental Figures

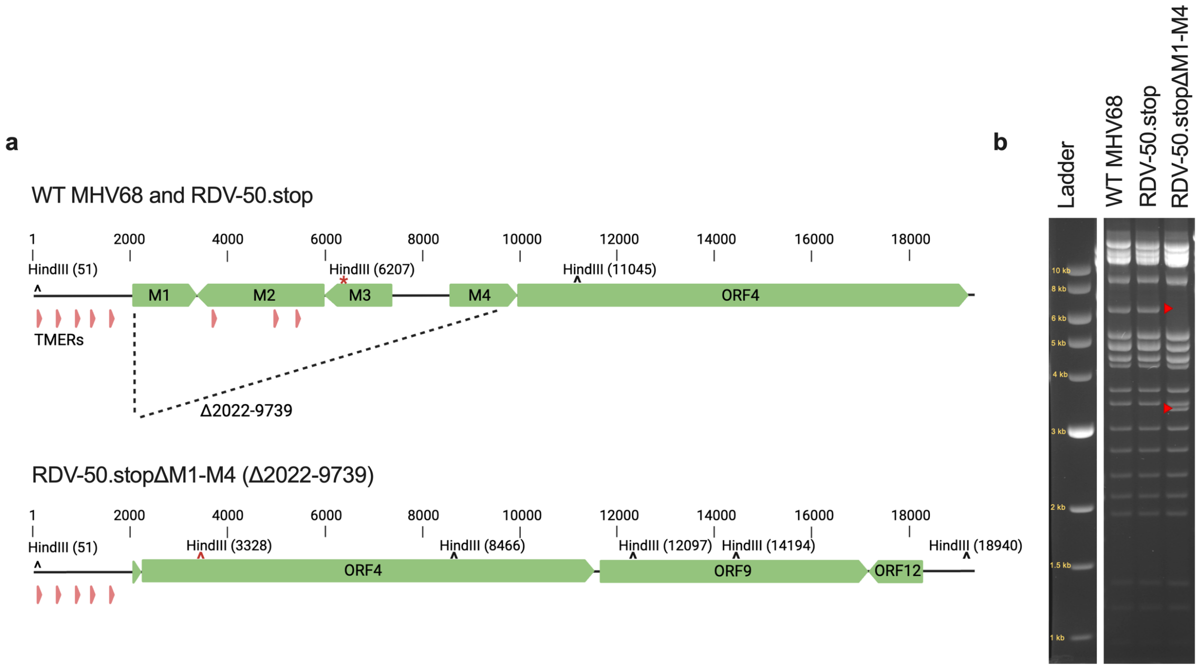

Supplementary Figure 1 Generation of recombinant RDV-50.stopΔM1-M4 MHV68 upon deletion of unique M gene locus. (a) Schematic describing the left end of WT MHV68 and the parental RDV-50.stop. M1- M4 genes and TMERs 6-8 (nucleotides 2022 to 9739) were deleted from RDV-50.stop to generate MHV68 RDV-50.stopΔM1-M4. '*' and '^' denote HindIII digestion sites. '*' indicates the site lost with the deletion of 2022 to 9739 bp. Figure made with BioRender. **(b)** In the HindIII restriction digest, WT MHV68 and RDV-50.stop has the expected 6156 bp fragment, while the RDV-50.stopΔM1-M4 genome has the expected loss of the 6156 bp fragment and gain of a 3277 bp fragment, indicated by red arrows.

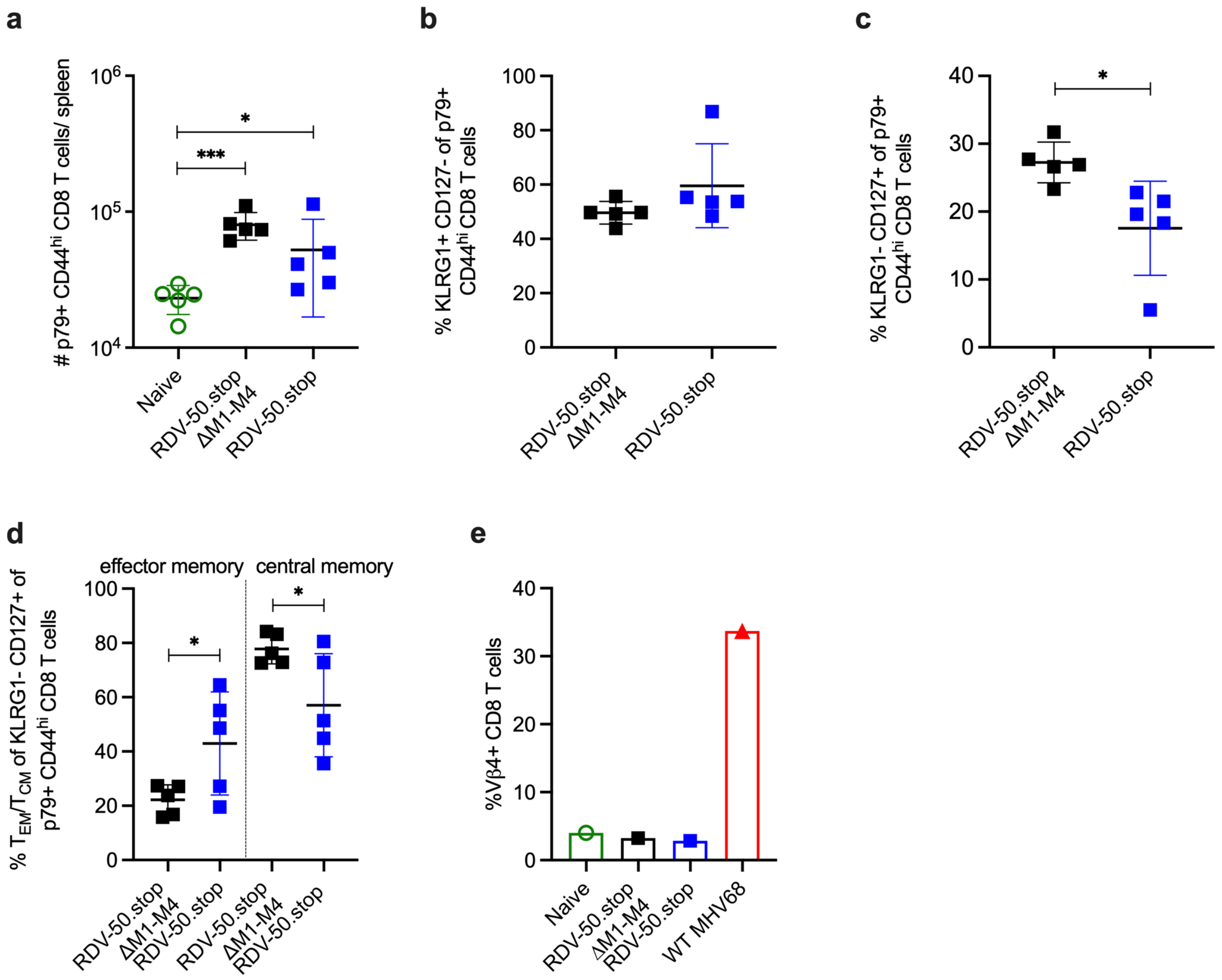

Supplementary Figure 2 RDV-50.stopΔM1-M4 generates virus-specific CD8 T cell responses but does not induce M1 driven Vβ4+ CD8 T cells upon a prime-boost regimen in C57BL/6 mice. C57BL/6 mice were either sham-vaccinated or prime-boost vaccinated IP with 1x10^6 PFU RDV-50.stopΔM1-M4 or RDV-50.stop. Naïve mice were age-matched, non-vaccinated controls. **(a)** Total p79-tetramer+ CD8 T cells per spleen of individual mice at d28 post-boost. Percentage of p79-dextramer+ CD8 T cells with markers of **(b)** short-lived effector cell (SLEC, KLRG1+CD127-) and **(c)** memory precursor effector cell subsets (MPEC, KLRG1-CD127+). **(d)** MPECs were further delineated into CD62L- effector and CD62L+ central MPECs for p79- dextramer+ CD8 T cells. **(e)** Percentage of Vβ4+ CD8 T cells in pooled spleens of mice with RDV-50.stop or RDV-50.stopΔM-M4 vaccination or infection with WT MHV68. For **(a-d)**, symbols represent individual mice, (N=5), and for **(e)** symbols represent pooled spleens from 5 mice.; bars and whiskers are mean +/- SD. *, p<0.05; **, p<0.01; ***, p<0.001 in Sidak's multiple comparisons test of one-way ANOVA **(a)**; in two-tailed unpaired t test **(b-d)** between the indicated groups.

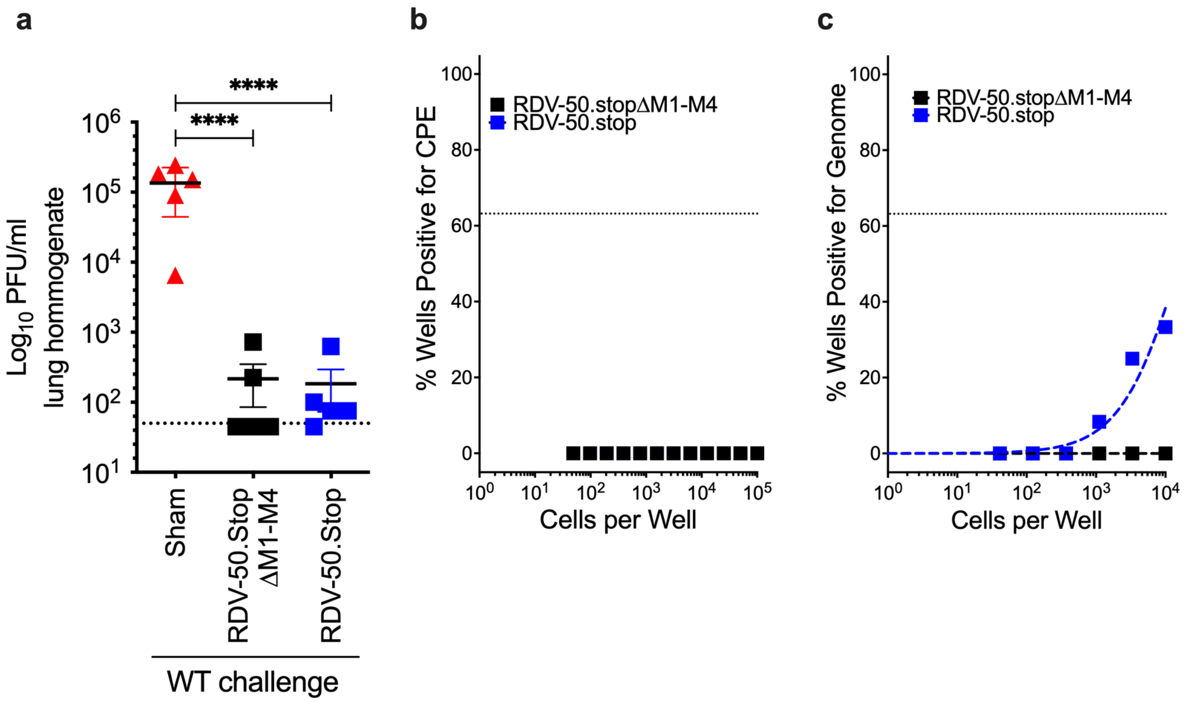

Supplementary Figure 3 Vaccination with RDV-50.stopΔM1-M4 reduces acute replication, latency and reactivation in C57BL/6 mice. C57BL/6 mice either sham-vaccinated or prime-boost vaccinated IP with $1x10^6$ PFU of RDV-50.stopΔM1-M4 or RDV-50.stop followed by IP challenge with $5x10^3$ PFU of WT MHV68 at d28 post-boost. **(a)** Acute replication at d7 post-challenge determined by measuring infectious particles per ml lung homogenate. (N=5 mice per experiment); bars and whiskers represent mean +/- SD. ****, p<0.0001 in Sidak's multiple comparisons test of one-way ANOVA between the indicated groups. **(b)** The frequency of latency determined by limiting dilution nested PCR of intact splenocytes for the viral genome at day d28 post-boost. **(c)** The frequency of explant reactivation determined by limiting dilution coculture of intact viable splenocytes on a monolayer of primary MEFs d28 post-boost. Disrupted splenocytes plated in parallel did not detect preformed infectious virus in the vaccinated animals. For **(b-c),** symbols represent pool of five individual mice per experiment.

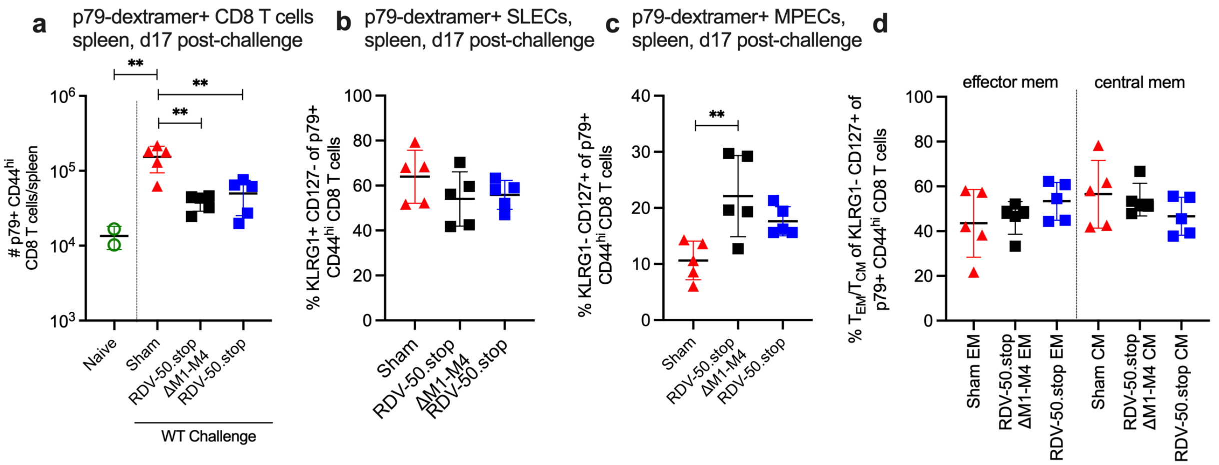

Supplementary Figure 4 Evaluation of T cell response to MHV68 in the spleens of vaccinated mice at seventeen days post-challenge with WT virus. C57BL/6 mice were either sham-vaccinated or prime-boost vaccinated IP with 1x10^6 PFU RDV-50.stopΔM1-M4 or RDV-50.stop followed by IN challenge with 1x10^3 PFU WT MHV68 at d28 post-boost and analyzed d17 post-challenge. **(a)** Total p79-dextramer+ CD8 T cells per spleen of individual mice with the indicated vaccination regimen. Percentage of p79-dextramer+ CD8 T cells with markers of **(b)** short-lived effector cell (SLEC, KLRG1$^+$CD127$^-$) and **(c)** memory precursor effector cell subsets (MPEC, KLRG1$^-$CD127$^+$). **(d)** MPECs were further delineated into CD62L$^-$ effector and CD62L$^+$ central MPECs for p79-dextramer+ CD8 T cells. For each graph, symbols represent individual mice, (N=3-5); bars and whiskers are mean +/- SD. **, p<0.01 in Sidak's multiple comparisons test of one-way ANOVA between the indicated groups.
